# Forecasting incidence of infectious diarrhea using random forest in Jiangsu Province, China

**DOI:** 10.1186/s12879-020-4930-2

**Published:** 2020-03-14

**Authors:** Xinyu Fang, Wendong Liu, Jing Ai, Mike He, Ying Wu, Yingying Shi, Wenqi Shen, Changjun Bao

**Affiliations:** 1 School of Public Health, Nanjing Medical University, Nanjing, 211166 China; 2 Jiangsu Provincial Center for Disease Control and Prevention, Nanjing, 210009 China; 3 NHC Key laboratory of Enteric Pathogenic Microbiology, Nanjing, 210009 China; 4 Mailman School of Public Health, Columbia University, New York, NY 10027 USA

**Keywords:** Infectious diarrhea, Forecasting, Random forest

## Abstract

**Background:**

Infectious diarrhea can lead to a considerable global disease burden. Thus, the accurate prediction of an infectious diarrhea epidemic is crucial for public health authorities. This study was aimed at developing an optimal random forest (RF) model, considering meteorological factors used to predict an incidence of infectious diarrhea in Jiangsu Province, China.

**Methods:**

An RF model was developed and compared with classical autoregressive integrated moving average (ARIMA)/X models. Morbidity and meteorological data from 2012 to 2016 were used to construct the models and the data from 2017 were used for testing.

**Results:**

The RF model considered atmospheric pressure, precipitation, relative humidity, and their lagged terms, as well as 1–4 week lag morbidity and time variable as the predictors. Meanwhile, a univariate model ARIMA (1,0,1)(1,0,0)_52_ (AIC = − 575.92, BIC = − 558.14) and a multivariable model ARIMAX (1,0,1)(1,0,0)_52_ with 0–1 week lag precipitation (AIC = − 578.58, BIC = − 578.13) were developed as benchmarks. The RF model outperformed the ARIMA/X models with a mean absolute percentage error (MAPE) of approximately 20%. The performance of the ARIMAX model was comparable to that of the ARIMA model with a MAPE reaching approximately 30%.

**Conclusions:**

The RF model fitted the dynamic nature of an infectious diarrhea epidemic well and delivered an ideal prediction accuracy. It comprehensively combined the synchronous and lagged effects of meteorological factors; it also integrated the autocorrelation and seasonality of the morbidity. The RF model can be used to predict the epidemic level and has a high potential for practical implementation.

## Background

Infectious diarrhea is one of the major causes of morbidity and mortality in infants and younger populations. It is a major global public health issue, particularly in developing countries [1]. In 2015, diarrheal diseases led to an estimated 688 million illnesses and 499,000 deaths among children under the age of 5 [2]. Over the past decade, morbidity has also increased in various regions in China [3]. Thus, an accurate forecast of infectious diarrhea based on predictive models is crucial for public health authorities to clearly understand its epidemic characteristics, track seasonal updates in advance, and select the main response actions such as the surveillance of disease and deployment of emergency supplies [4].

The autoregressive integrated moving average (ARIMA) model has been widely used as classical method for diarrhea incidence prediction, however, it has some limitations at the same time [4–7]. For example, Yang et al. [4] used the ARIMA model without climate terms in an early warning systems for diarrhea but achieved a poor fit. Several studies have reported that meteorological factors are associated with diarrhea and can be used to predict its incidence [8, 9]. Yan et al. [7] developed a multivariable ARIMA (ARIMAX) model considering temperature and rainfall but only achieved high short-term predictive accuracy, possibly because the ARIMAX model assumed linear relationships between the independent and dependent variables. However, meteorological factors have been reported to be non-linearly associated with the infectious diarrhea epidemic [9, 10].

The RF model is a new regression method and can address the limitations of ARIMA/X models in the prediction of diarrhea incidence [11–14]. It can effectively extract non-linear relationships from data. The RF model uses independent variables to create classification and regression trees (CARTs), wherein each constituent tree is trained on a potentially non-linear regression space. The RF model may achieve predictive stability in terms of the actual instable morbidity. Using the RF model, the training set for each tree is randomly selected from the data, and the final predicted value is the average of all CART outputs. RF model has been widely used for infectious-disease prediction such as West Nile virus infection and Bovine viral diarrhea [12, 13]. Notably, Michael et al. [14] reported that an RF model has advantages over the ARIMA model in predicting avian influenza H5N1 outbreaks. However, no studies have used an RF model to predict the incidence of infectious diarrhea .

This study was aimed at developing an optimal RF model for predicting infectious diarrhea epidemics with meteorological factors in Jiangsu Province, China. Meanwhile, the performance of the RF model was compared with those of the ARIMA/X models. The model can be used to develop an early warning system for infectious diarrhea to facilitate preventive strategies in a more effective manner.

## Methods

### Study area

Jiangsu Province, located along the eastern-coast of China (latitude 30°45′-35°20′N and longitude 116°18′-121°57′E), has an area of 102,600 km^2^ and a population of approximately 80 million. It has a typical temperate subtropical monsoon climate with mild temperature, moderate rainfall and a distinct four-season pattern.

### Data sources

In China, infectious diarrhea (excluding cholera, dysentery, typhoid and paratyphoid) is an intestinal infectious disease with diarrhea and/or vomiting as the main symptom. It has been listed as a legal Class C infectious disease [3]. An infectious diarrhea case, clinically diagnosed or etiologically confirmed by any hospital or healthcare institution throughout the country, must be reported timely and directly to the National Notifiable Disease Surveillance System (NNDSS) [15] (http://www.cdpc.chinacdc.cn). In this study, the weekly numbers of infectious diarrhea cases in Jiangsu Province during 2012–2017 were downloaded from the NNDSS, including both clinically diagnosed and etiologically confirmed cases.

The demographic data were collected from the Jiangsu provincial statistics department. The weekly meteorological factors were calculated based on the daily data obtained from the Jiangsu Meteorological Service Center. The data included atmospheric pressure, mean temperature, maximum temperature, minimum temperature, precipitation, relative humidity and sunshine duration.

### ARIMA/X model

ARIMA model, namely the Box−Jenkins model, has been widely used for time series analysis [16]. The seasonal ARIMA, that incorporates seasonal variation based on ARIMA model, performs better in the presence of clear seasonal patterns [17, 18]. It is denoted as ARIMA(p,d,q)(P,D,Q)_s_, where p, d and q indicate the orders of general auto-regression (AR), differencing and moving average (MA) terms; P, D and Q are the orders of seasonal AR, differencing and MA terms, respectively; and s is the seasonal periodicity (s = 52 weeks in this study) [18].

The fitting of the ARIMA model involves the following three essential steps:

First, an augmented Dickey−Fuller test is conducted to detect whether the original time series is stationary (statistical properties such as the mean and variance are all constant over time). If not, a logarithmic transformation or difference is adopted to achieve stability.

Second, ARIMA models are established for a stationary time series, and the model with the minimum Akaike information criterion (AIC) and Bayesian information criterion (BIC) values is considered the optimal model. The model parameters are then estimated using the conditional least squares method.

Third, to verify the adequacy of the ARIMA model, a Box−Ljung test is conducted to check whether the residual series is a white noise sequence. A white noise sequence is a purely random time series without an autocorrelation, and useful information cannot be extracted from the sequence for model fitting. If not, the model must be reestablished. Finally, a prospective prediction is conducted using the optimal model.

Based on the optimal ARIMA model, a multivariate ARIMA model including meteorological factors as external regressors [19] is further developed, and is referred to as the ARIMAX model.

In this study, the ARIMA/X models were used as references to evaluate the performance of the RF model. A cross-correlation analysis was used to identify the lagged associations (1–4 week lag [20, 21]) between the meteorological factors and the incidence of infectious diarrhea.

### RF model

RF model is an ensemble machine learning method proposed by Breiman [11]. It creates multiple CARTs, wherein each tree is trained on a bootstrap sample of the original training data using a randomly selected subset of input variables, and taking the average outputs of the CARTs as the final prediction. One of its most important features is the calculation of the variable importance, which measures the association between a given variable and the accuracy of the prediction, based on the percentage of increase in the mean square-error (%IncMSE).

The RF model fitting consists of four essential steps [14]:

First, a bootstrap sampling method is used to randomly select sample units from the original training data to create multiple CARTs.

Second, the bootstrap sampling method is used again to select the candidate variables for each CART. In this study, the related meteorological variables were chosen as the predictors. Meanwhile, the 1–4 week lag morbidity and time variable were incorporated into the RF model to consider the effects of autocorrelation and seasonality of the dependent variable, respectively.

Third, the average outputs from all CARTs are calculated as the final predictive value.

Fourth, the importance of each variable is assessed based on the reduction in accuracy.

### Model evaluation

Three models were fitted during this study, namely an RF model with meteorological factors, a univariate ARIMA model and a multivariate ARIMAX model. The data subset for the period of 2012–2016 was used as the training set to fit the models, and data from 2017 were used as the test set to evaluate the forecasting accuracy. The root mean square error (RMSE) and mean absolute percentage error (MAPE) were selected to evaluate the performance of each model; they were calculated as follows:

$$ RMSE=\sqrt{\frac{\sum_{t=1}^n{\left({\hat{y}}_t-{y}_t\right)}^2}{n}} $$

$$ MAPE=\frac{1}{n}\sum \limits_{t=1}^n\frac{\left|{\hat{y}}_t-{y}_t\right|}{y_t} $$

where n is the number of real data or predicted values, *y*_*t*_ is the real data, and ${\hat{y}}_t$ is the predicted value.

### Statistics analysis

All analyses were conducted in R (version3.5.1). A seasonal decomposition was conducted to elucidate the temporal pattern of infectious diarrhea. The RF model was fitted using the “randomForest” package, and the ARIMA/X models were fitted using the “Forecast” package.

## Results

### General description

**Fig. 1:**
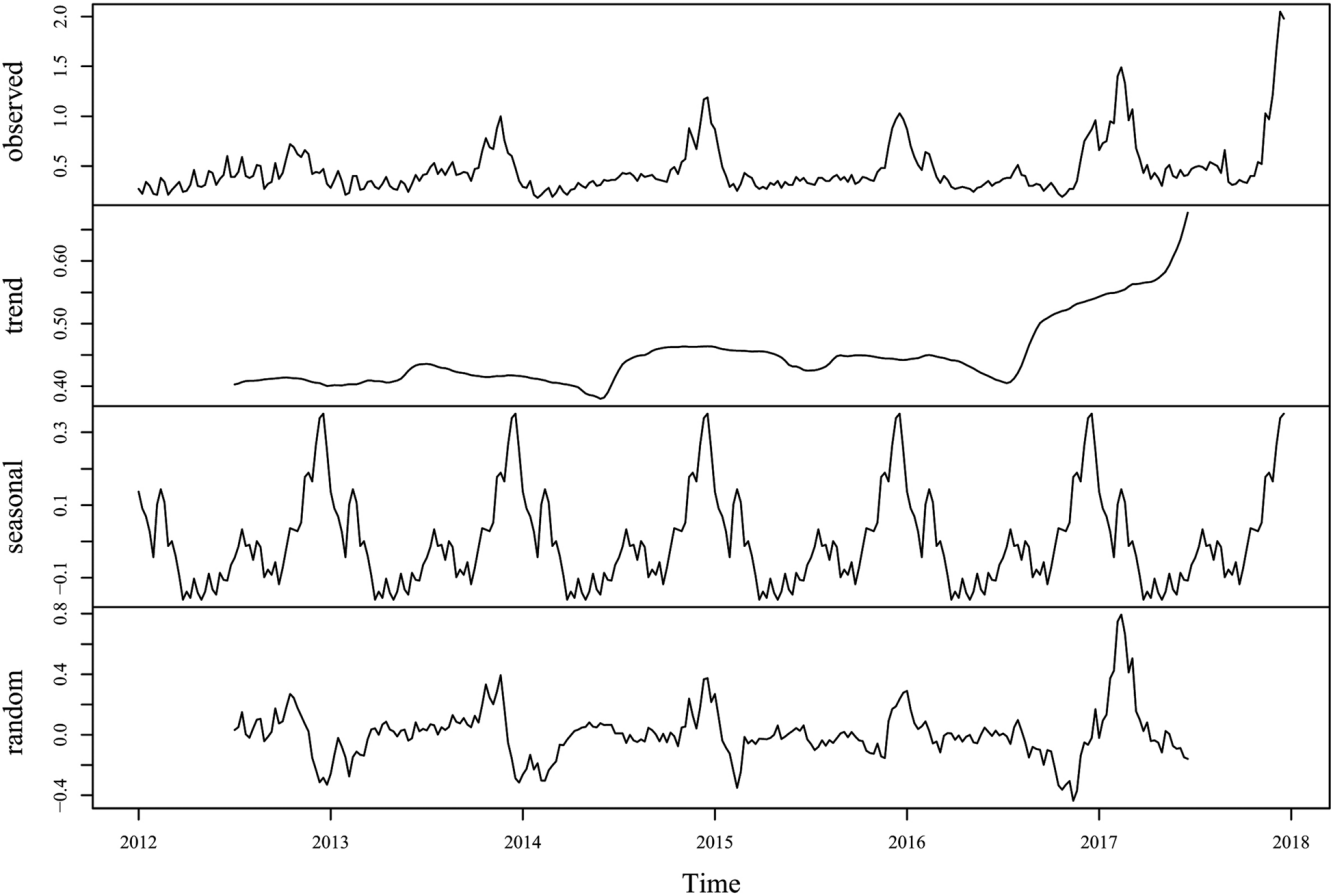
Weekly observed cases of infectious diarrhea in Jiangsu Province, 2012–2017. Note: From top to bottom, the lines represent actual observations, the trend, seasonal, and random components

A total of 102,020 cases were detected during 2012–2017 in Jiangsu Province, China, reaching an annual average incidence of 21.40 per 100,000. As shown in Fig. 1, the incidence exhibited an increasing long-term trend during these 6 years. Moreover, a distinct seasonality was exhibited, i.e., two incidence peaks were observed during each year: namely higher winter peak from December to February and a lower summer peak from July to September. The descriptive statistics for the meteorological factors were summarized in Table 1.

**Table 1:** Summary of weekly meteorological factors in Jiangsu Province, 2012–2017

Variable	Min	P25	Median	P75	Max
Atmospheric pressure (Pa)	998.58	1007.02	1015.38	1022.56	1032.09
Mean temperature (°C)	−2.19	7.39	17.13	23.67	32.65
Maximum temperature (°C)	1.25	12.36	22.38	27.60	37.41
Minimum temperature (°C)	−4.77	3.59	13.08	20.63	28.24
Relative humidity (%)	45.93	68.06	74.69	80.40	91.88
Precipitation (mm)	0.00	3.53	11.94	30.12	59.66
Sunshine duration (h)	2.25	27.71	37.50	48.72	82.01

### Correlation analysis

As presented in Table 2, the atmospheric pressure and precipitation were significantly associated with 0–2 week and 0–3 week lag morbidity, respectively. Meanwhile, the relative humidity was related to the synchronous morbidity (r_s_ = − 0.13, *P* = 0.02). The temperature variables and sunshine duration were not correlated with the incidence.

**Table 2:** Cross correlation coefficients between infectious diarrhea and meteorological factors in Jiangsu Province, 2012–2017

Lag	Atmospheric pressure (Pa)	Mean temperature (°C)	Maximum temperature (°C)	Minimum temperature (°C)	Relative humidity (%)	Precipitation (mm)	Sunshine duration (h)
0	0.21**	−0.10	−0.09	−0.11	−0.13*	−0.23**	0.07
1	0.17**	−0.06	−0.06	−0.07	−0.08	−0.22**	0.05
2	0.12*	−0.02	−0.01	−0.02	−0.04	−0.14*	0.03
3	0.08	0.03	0.03	0.03	−0.02	−0.12*	0.04
4	0.04	0.08	0.08	0.08	0.04	−0.08	0.05

Note: *P < 0.05, **P < 0.01

### Model fitting

#### ARIMA/X model

The original time series of the incidence of infectious diarrhea was stationary (Dickey−Fuller = −4.26, *P* < 0.01). Univariate ARIMA models were developed. The best-fitting ARIMA model was determined to be ARIMA (1,0,1)(1,0,0)_52_, with a minimum AIC = − 575.92 and a minimum BIC = − 558.14. The Ljung−Box test results suggested that the residual series of the model was a white noise sequence (*χ*^*2*^ = 0.01, *P =* 0.93).

Next, related meteorological factors were added as covariates into the optimal ARIMA model to establish the multivariate ARIMAX models. Finally, ARIMAX (1,0,1)(1,1,0)_52_ with 0–1 week lag precipitation was identified as the optimal ARIMAX model, with a minimum AIC of − 578.58 and a minimum BIC of − 578.13 (Ljung−Box test: *χ*^*2*^ = 0.00548, *P* = 0.10).

RF model.

**Fig. 2:**
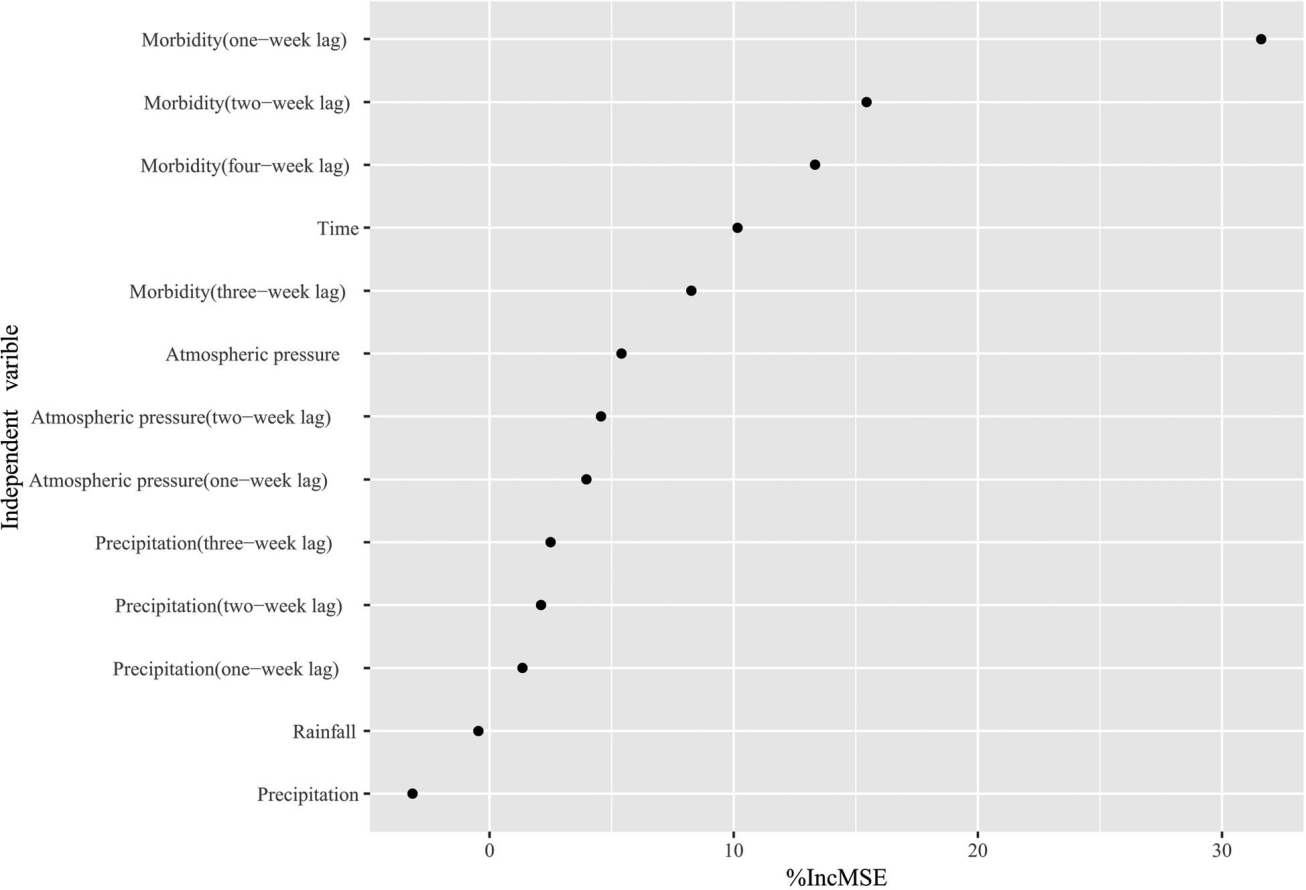
Variable importance in random forest regression model for infectious diarrhea

An RF model was constructed using atmospheric pressure, precipitation and their lagged terms, relative humidity, 1–4 week lag morbidity and time variable as predictors. Figure 2 indicated that the lag dependent terms were the most imperative among all the applied predictors. The atmospheric pressure and its lagged terms were the most vital meteorological factors, followed by a lag in precipitation.

### Prediction performance comparison

**Fig. 3:**
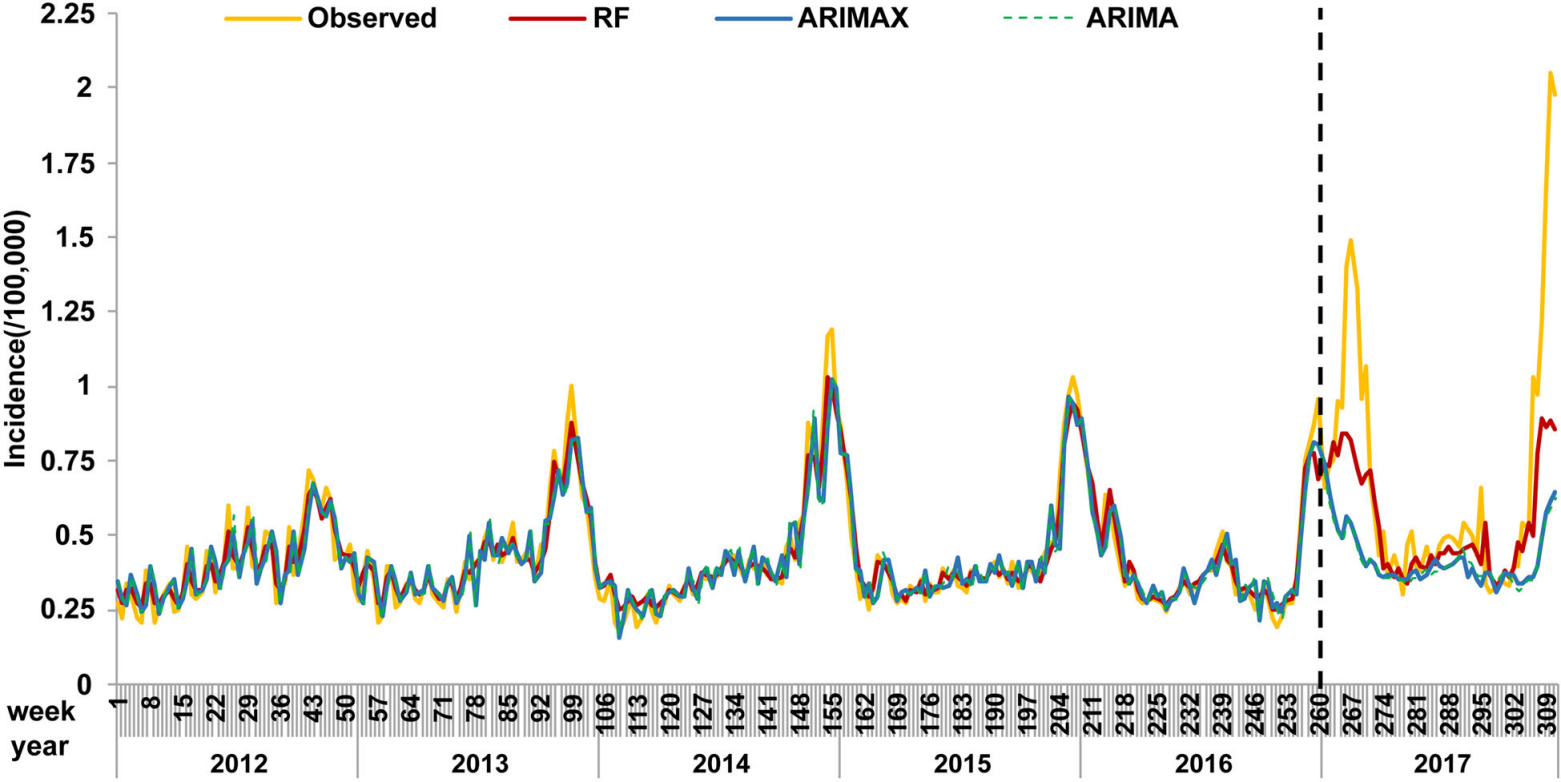
Observed infectious diarrhea incidences and values predicted by different models. Note: The left side of the vertical line indicates the model fitting stage, and the right side indicates the prospective stage

Table 3 compared the RF and ARIMA/X models, the predictive outputs of which were reported in Fig. 3. The RF model with meteorological factors outperformed the ARIMA/X models in both model fitting and prospective stages in terms of RMSE and MAPE. The values predicted by the RF model matched the actual values very well, with a MAPE of approximately 20%. The performance of the ARIMAX model was comparable to that of the ARIMA model with a high MAPE of approximately 30%.

**Table 3:** Performance of the RF and ARIMA/X models

Model	RMSE		MAPE (%)	
	Training set	Testing set	Training set	Testing set
RF	0.04	0.31	6.88	20.89
ARIMAX(1,0,1)(1,0,0)_52_	0.08	0.46	13.64	28.06
ARIMA(1,0,1)(1,0,0)_52_	0.08	0.45	13.78	28.53

## Discussion

The incidence of infectious diarrhea in Jiangsu Province exhibited a long-term gradual growth trend. Mathematical prediction models are urgently required to reinforce integrated management to monitor, control and prevent infectious diarrhea. We constructed an RF model with meteorological factors, which delivered a good accuracy in predicting the incidence of infectious diarrhea with a MAPE of approximately 20%. It can relatively estimate the seasonal fluctuation of this disease well. The model may be used as an important tool by public health authorities.

The RF model is more suitable than the ARIMA/X method for predicting an infectious diarrhea epidemic within the study region. The performance of the ARIMAX model was comparable to that of the ARIMA model, which suggested that the introduction of meteorological factors did not significantly optimize the prediction accuracy of the ARIMA model. This finding was consistent with the findings of other previous studies [3–5]. The RF model provided a meaningfully better fit to the data in terms of RMSE and MAPE. Compared with the ARIMA/X models, the prediction error of the RF model decreased by approximately 50 and 30% in the training and testing sets, respectively. This is because the RF model can better fit non-linear relationships. Moreover, compared with the ARIMAX model, the RF model is not influenced by the multicollinearity, mainly because of the random selection of variables for each tree in the RF [11]. The meteorological factors and their lagged terms were incorporated into the models when they significantly correlated with the morbidity. All of them exhibited a certain degree of importance, which suggested that the RF model comprehensively combined the climatic variables and their lagged effects. In particular, the models partly underestimated the incidence of infectious diarrhea in 2017. This is primarily due to the sharp increase in morbidity in 2017, which indicated that the potential influencing factors might have changed over a 52-week period, such as increase in the number of outbreaks, or changes in the pathogen spectrum [22, 23]. In addition to meteorological factors, some other variables should be considered to better optimize the prediction accuracy of the RF model.

Atmospheric pressure, precipitation, and relative humidity were all correlated with the incidence of infectious diarrhea in Jiangsu Province with 0–2 week, 0 week and 0–3 week lag, respectively. However, Tao et al. [20] reported that the atmospheric pressure and relative humidity were related to the 0–1 week lag diarrhea morbidity in Lanzhou city (northwest China). The relative humidity was related to 4-week lag in the incidence of diarrhea in Beijing city (north China) [21]. This difference may be due to the regional differences in pathogen composition and climatic conditions. Furthermore, the meteorological factors significantly contributed to the forecasting ability of the RF model, with atmospheric pressure as the main contributor. Potential mechanisms can include the influencing pathogen survival and air barrier. A high atmospheric pressure may be conducive to the survival of infectious diarrhea causing microorganisms, such as the rotavirus, in the environment [24]. A high atmospheric pressure can hinder the airflow and serve as a barrier to the spreading of airborne pathogens thereby increasing their concentration at a smaller scale, which may lead to more diarrhea infection [25]. The precipitation had a moderate importance in the RF model, particularly the 3-week lag effect. This implied that the precipitation during the previous 3 weeks may influence the morbidity and can thus be used in its prediction. The relative humidity was identified as the least important factor. The relative humidity in Jiangsu Province exhibited a narrow variation at the weekly level, and did not fit well with the morbidity. These findings may help future studies in analyzing the specific relationship between the climate and infectious diarrhea.

Notably, the prediction performance is likely to vary in different climatic regions. The generalizability of the RF model for the incidence of infectious diarrhea in Jiangsu Province to other regions might not be straightforward. However, the use of the RF model incorporating meteorological factors in the detection and prediction of infectious diarrhea may provide an opportunity for reallocating healthcare resources more efficiently in other regions. In addition, considering the autocorrelation and clear seasonality of infectious diarrhea, the 0–4 week lag morbidity and time variable were incorporated into the RF model and were more important than the meteorological factors in improving the prediction accuracy of the RF model. These strategies should be used as a reference when fitting similar RF models.

This study had a few limitations. First, some mild cases may use home therapies, and cases with atypical symptoms may be misdiagnosed, therefore, the reported data may underestimate the level of morbidity. Second, only meteorological factors were considered to improve the prediction ability. Other factors associated with infectious diarrhea may also be used as good predictors and should be studied further. Third, similar to other machine learning methods such as artificial neural networks, the RF model cannot explain the specific non-linear relationship between meteorological factors and the disease.

## Conclusions

The RF model with meteorological factors demonstrated a satisfactory prediction accuracy and can be used to predict the epidemic level, demonstrating its potential and practical applicability. The autocorrelation and seasonal variation of the dependent variables are crucial for the prediction model. In addition, the synchronous effects of meteorological factors and their cumulative effects over a period of time were combined to improve the model. Future studies should be conducted to explore an RF model with meteorological and other variables for the development of a useful tool for predicting other major infectious diseases.

## Data Availability

The datasets used in this study are available from the corresponding author on reasonable request.

## Abbreviations

ARIMA: Autoregressive integrated moving average
RF: Random forest
MAPE: Mean absolute percentage
CART: classification and regression tree
AR: Auto-regression
MA: Moving average
AIC: Akaike information criterion
BIC: Bayesian information criterion
RMSE: Root mean square error
